# Impact of Cancerous Pulmonary Involvement on Outcomes in COVID-19 Patients

**DOI:** 10.7759/cureus.37671

**Published:** 2023-04-17

**Authors:** Atif Saleem, Maria Qubtia, Dawood Misbah, Maham Majid, Arsalan Zeb, Fattahullah Khan

**Affiliations:** 1 Internal Medicine, Shaukat Khanum Memorial Cancer Hospital and Research Centre, Peshawar, PAK; 2 Medical Oncology, Shaukat Khanum Memorial Cancer Hospital and Research Centre, Peshawar, PAK; 3 Medical Oncology, Mater Private Hospital, Dublin, IRL; 4 Oncology, Shaukat Khanum Memorial Cancer Hospital and Research Centre, Peshawar, PAK

**Keywords:** pulmonary metastasis, cancer, sars-cov-2, pulmonary cancer, covid-19 in cancer patients

## Abstract

Background: SARS-CoV2 is a highly contagious virus causing COVID-19 (Corona virus disease 2019), which has resulted in more than 6 million deaths worldwide as of June 2022. Mortality in COVID-19 has mainly been attributed to respiratory failure. Previous studies showed that the presence of cancer did not adversely affect the outcome of COVID-19. However, in our clinical practice, it was noted that in cancer patients with pulmonary involvement, COVID-19-related morbidity, and morbidity were high. Therefore, this study was designed to assess the impact of cancerous pulmonary involvement on COVID-19 outcomes and to compare clinical outcomes of COVID-19 in cancer and non-cancer population, with further discretion between cancers with and without pulmonary involvement.

Methods: We performed a retrospective study from April 2020 until June 2020 with a sample size of 117 patients with a confirmed diagnosis of SARS-CoV2 on nasal swab PCR. Data was extracted from HIS (Hospital Information System). Hospitalization, supplemental oxygen, ventilatory support, and death were compared between non-cancer and cancer patients with a particular focus on pulmonary involvement.

Results: Admissions, supplemental oxygen requirement, and mortality were significantly higher in cancer patients with pulmonary involvement (63.3%, 36.4%, and 45%, respectively) compared to cancer patients without pulmonary involvement (22.1%, 14.7%, and 8.8% respectively) (p-values: 0.00003, 0.003, and 0.00003 respectively). In the non-cancer group, there was no mortality, only 2% required admission, and none needed supplemental oxygen.

Conclusion: We conclude that the cancer patient with pulmonary involvement was significantly at higher risk of complications and death from COVID when compared with the non-pulmonary cancer group and the general population.

## Introduction

The COVID-19 pandemic, caused by the coronavirus, has significantly affected healthcare practices worldwide since its discovery in 2019. As of June 2022, there have been more than 534 million cases of COVID-19 infections, resulting in more than 6.3 million deaths [1]. It spreads mainly by respiratory droplets entering the upper respiratory airways. Hence upper respiratory symptoms like the common cold, stuffy nose, anosmia, headaches, sore throat, and fever are common. However, like other SARS (severe acute respiratory syndrome), it can involve the gastrointestinal and lower respiratory tract, where it further replicates. The body's response to this virus is variable and complex. It could range from mild to moderate self-limiting illness, seen in the majority, to life-threatening and fatal conditions depending on the viral strain, loads, the resulting body's immune response, and comorbidities like hypertension and diabetes [2-4].

The diagnosis is based on symptoms, contact history, laboratory confirmation with PCR assay, and in some cases, radiological findings [5]. The pathogenesis of Covid-19 is not clearly understood. However, it is hypothesized that a dysregulated immune system and cytokine storm in critically ill patients are suspected to be the main culprits. The immune response to the virus seems crucial to this infection's severity and poor outcome [6,7]. Respiratory involvement is associated with the worst outcome in the array of symptoms. Therefore, those patients with poor lung reserves would be more prone to severe infection with dismal outcomes. Also, in the cancer population, pulmonary functions are compromised because of primary or metastatic lung involvement, anatomical or physiological deficit resulting from oncological intervention, and comorbidities [8,9]. Moreover, studies suggest that patients with lung cancer have higher morbidity and mortality compared to other cancer and non-cancer patients [10-15].

Patients with associated comorbidities and factors such as age, male gender, and smoking were at higher risk of increased mortality and morbidity from COVID-19 [6,16-18]. Similarly, patients with metastatic disease had adverse outcomes regarding death rate, ICU admission, and use of invasive mechanical ventilation [19-20].

Studies have been conducted worldwide to compare the clinical outcomes in COVID-19 patients with & without cancers as well as between pulmonary and non-pulmonary cancers [21-22]; however, such a study has yet to be conducted in Pakistan. In our setting, we noted that patients with pulmonary involvement were more prone to adverse outcomes of COVID-19 compared with those without pulmonary involvement and the general population. Our study aims to compare the outcome of COVID-19 in cancer and non-cancer patients, along with a comparison between pulmonary and non-pulmonary cancers.

## Materials and methods

This was a retrospective, closed-ended study conducted on patients with or without cancer diagnosed with COVID-19 from April 1st, 2020, to June 30th, 2020, at our Shaukat Khanum Memorial Cancer Hospital and Research Center, Peshawar, Pakistan. The data was analyzed in June 2021, and any changes in a health condition or COVID-19-related outcomes during the follow-up period were noted. We aimed to look at the impact of cancer in general, and pulmonary involvement in particular, on the outcome of COVID-19 compared with the general population. Participants must be diagnosed with coronavirus at some point, having a laboratory confirmation of SARS-CoV-2 infection on a nasopharyngeal swab. Of note, as our center is a dedicated cancer hospital, therefore, the healthy individuals studied were hospital employees. We reviewed records of 117 patients extracted from HIS (Hospital information system), a system in which all patient data is saved. We included individuals diagnosed with COVID-19 through a laboratory confirmation of SARS-CoV2 infection on nasal swabs using a validated assay (PCR). We excluded clinically suspected COVID-19 without laboratory confirmation and pediatric patients. Being a retrospective study without direct contact with cancer patients, consent was not required. However, employees who were willing to volunteer were requested to sign an informed written consent which included necessary information on the purpose of the study, duration of participation, and the risks and benefits the study will have on the participants and the society, as per the IRB (Institutional Review Board) requirements. Participants were also made aware that their medical records were to be accessed throughout the period given; however, their information would remain confidential and confined to the study.

Patients' data and medical information were accessed through our Hospital Information System through their medical record number, a hospital-specific identification number for each patient. The data being collected was completely anonymized to maintain confidentiality. After that, the data was extracted for baseline characteristics which were gender, age (in years), comorbidities (diabetes, hypertension, congestive heart failure, ischemic heart disease, hypercholesterolemia, COPD, asthma), cancer diagnosis, pulmonary involvement by cancer, localized or metastatic disease. Clinical records were reviewed for the management offered to these patients with COVID-19. Hence, the management parameters of a requirement for hospitalization, oxygen supplementation and ventilation, and outcome of illness were noted for all the patients. The data was compiled and analyzed using SPSS v 26 to study the impact of comorbidities, cancer, and pulmonary involvement on the clinical outcomes, including hospitalization, oxygen supplementation, ventilation, and death. Chi-square was used to calculate the p-value, and a p-value equal to or less than 0.05 was considered significant.

## Results

Most patients were male (n=81, 69.2% vs. female: n=36, 30.8%). The median age was 47 years, ranging from 18 to 76 years. Most of the patients had no comorbidities (n=90, 76.9%). The non-cancer group comprised 38 patients (32.5%), whereas 79 patients (67.5%) were in the cancer group. Amongst patients with cancer, pulmonary involvement was present in 11 (14%) patients, while 68 (86%) patients had no pulmonary involvement. In addition, 25 (32%) patients in the cancer group had metastasis, whereas 54 (68%) had no metastasis (Table 1).

**Table 1:** Baseline characteristics of patients

Table 1: Baseline characteristics		n (%)
Gender	Male	81 (69.2)
	Female	36 (30.8)
Age (in years)		41.13 + 15.82
Comorbidities most common being hypertension and diabetes mellitus	Yes	27 (23.1)
	No	90 (76.9)
Cancer	Yes	79 (67.5)
	No	38 (32.5)
Pulmonary involvement	Not applicable (non-cancer)	38
	Yes (Primary and Mets)	11 (14)
	No	68 (86)
Metastatic	Not applicable (non cancer)	38
	Yes	25 (32)
	No	54 (68)

The number of patients requiring admission, supplemental oxygen, and ventilatory support was significantly higher in those having comorbidities (37%, 33.3%, and 11.1%, respectively) as compared to those with no comorbid conditions (14.4%, 5.6%, and 2.2% respectively) with respective p-values of 0.01, 0.0009 and 0.045. The total number of deaths was markedly higher in those having comorbidities (22.2 %) as compared to those with no comorbidities (5.6%), again with a significant p-value of 0.009 (Table 2-5).

**Table 2:** Impact of comorbidities on admission

		Admission required			p-value
		Yes (%)	No (%)	Total (%)	
Comorbidities	Yes	10 (37)	17 (63)	27 (100)	0.01
	No	13 (14.4)	77 (85.6)	90 (100)	

**Table 3:** Impact of comorbidities on requirement of supplemental oxygen

		Supplemental oxygen			p value
		Yes (%)	No (%)	Total (%)	
Comorbidities	Yes	9 (33.3)	18 (66.7)	27 (100)	0.00009
	No	5 (5.6)	85 (94.4)	90 (100)	

**Table 4:** Impact of comorbidities on requirement of ventilation (invasive or non-invasive)

		Ventilation (Invasive or non-invasive)			p value
		Yes (%)	No‎ (%)	Total (%)	
Comorbidities	Yes	3 (11.1)	24 (88.9)	27 (100)	0.045
	No	2 (2.2)	88 (97.8)	90 (100)	

**Table 5:** Impact of comorbidities on final outcome

		Final Outcome			p value
		Discharged (%)	Died (%)	Total (%)	
Comorbidities	Yes	21 (77.8)	6 (22.2)	27 (100)	0.009
	No	85 (94.4)	5 (5.6)	90 (100)	

**Figure 1:**
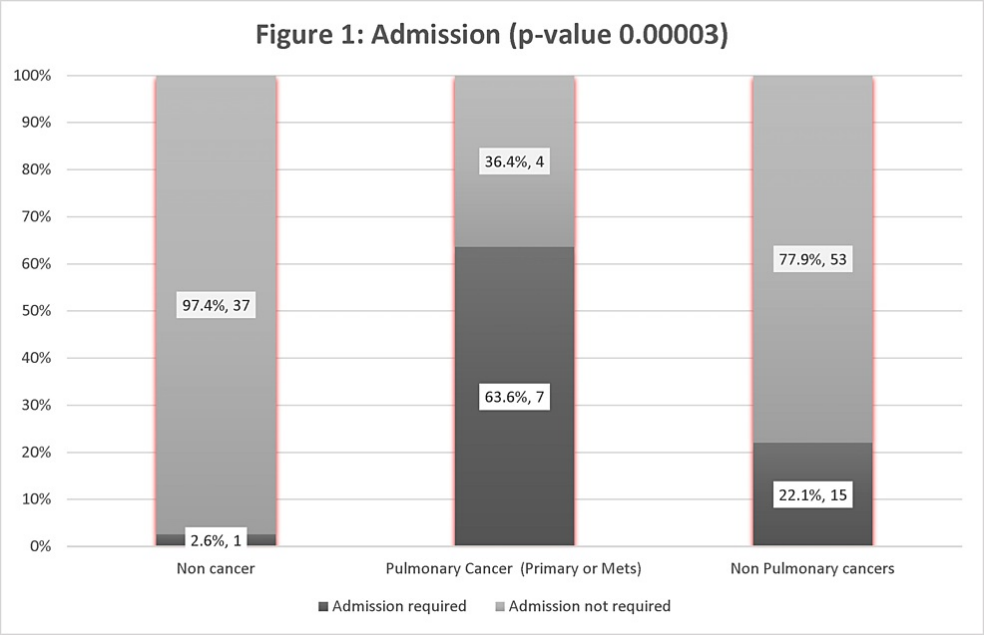
Admission requirement to pulmonary vs. Non-Pulmonary involvement.

Among cancer patients, the proportion of patients with pulmonary involvement requiring admission was significantly higher (63.6 %) than those with non-pulmonary cancers (22.1 %). In the non-cancer group, only one patient (2.6 %) required admission. The results were significant, with a p-value of 0.00003 (Figure 1).

**Figure 2:**
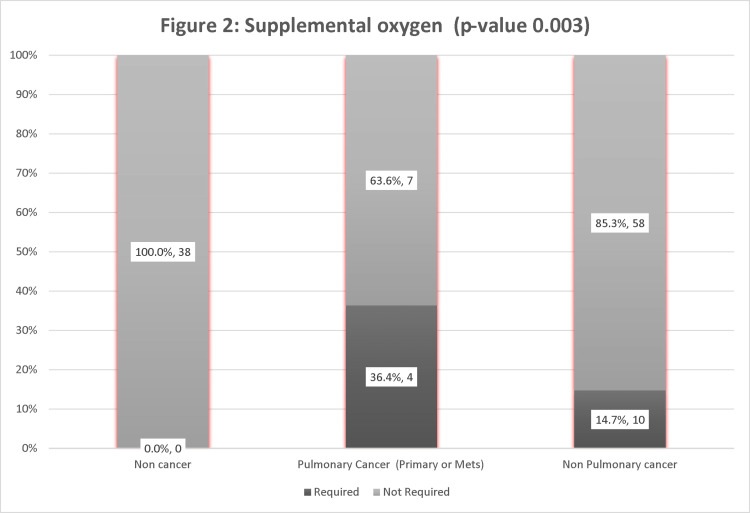
Supplemental oxygen requirement to pulmonary vs. Non-Pulmonary involvement.

In the cancer group, the proportion of patients with pulmonary involvement requiring supplemental oxygen was substantially more significant (36.4%) than those with non-pulmonary cancers (14.7 %). Whereas in the non-cancer group, none of the patients required supplemental oxygen. The results were significant, with a p-value of 0.003 (Figure 2).

**Figure 3:**
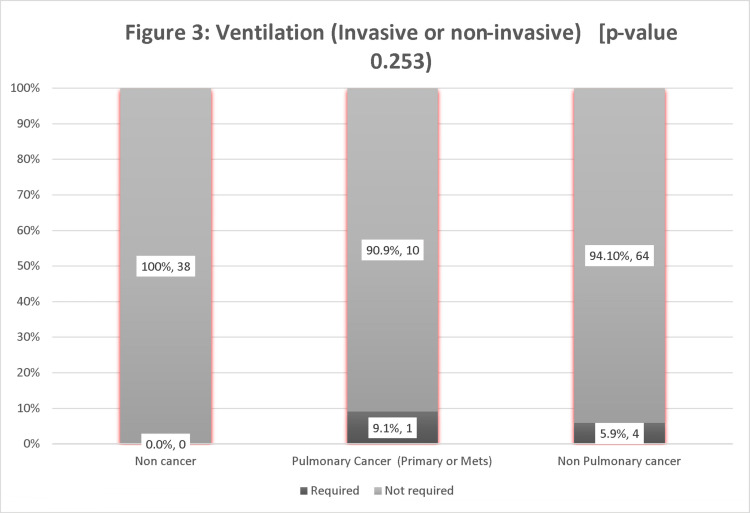
Ventilation requirement to pulmonary vs. Non-Pulmonary involvement.

Among the cancer patients, the proportion of patients with pulmonary involvement requiring ventilation was 9.1 % compared to 5.9% in the non-pulmonary cancer group. Whereas in the non-cancer group, none of the patients required ventilatory support. However, the p-value was insignificant (0.253) (Figure 3).

**Figure 4:**
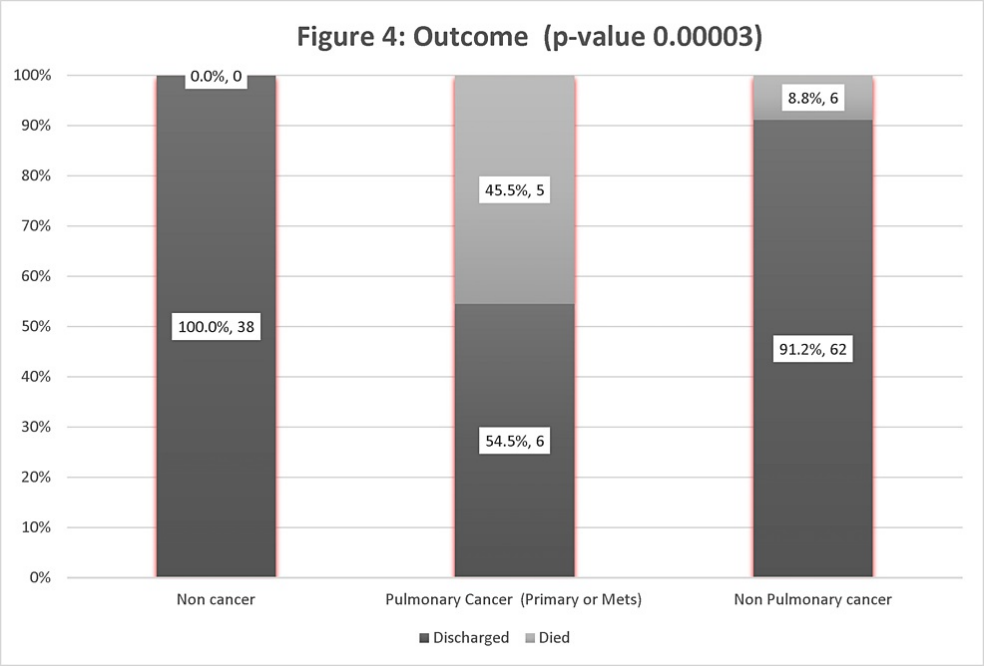
Outcome of pulmonary vs. Non-Pulmonary involvement.

In the cancer group, 45.5% died, compared to 8.8% with no pulmonary involvement. However, there were no deaths in the non-cancer group. Therefore, the p-value was significant at 0.00003 (Figure 4).

## Discussion

Similar to the global COVID-19 data observations, male preponderance was noted in our study. The median age (47 years) of affected COVID-19 patients in our study population was young, largely due to our region's low average life expectancy. Additionally, similar dynamics involve the early age at the time of cancer diagnosis. Thirdly, the comparison group of non-cancer individuals was the hospital staff in the pre-retirement age group.

This study observed that the patients with comorbidities such as hypertension and diabetes mellitus had higher hospitalization rates, oxygen and assisted ventilation requirement, and mortality. This trend confirmed what is already known about the behavior of this disease [6,17,23]. It can be contemplated from this observation that an appropriate, representative COVID-19 patient population was sampled.

It is evident from this study that the patients with cancer, specifically those with lung involvement, were more prone to develop severe covid pneumonitis requiring admission, supplemental oxygen, and ventilatory support [10-13]. These patients have poor lung reserves, and this increased propensity towards pulmonary decompensation increases the need for supplemental oxygen and, at times, ventilatory support. COVID-19, primarily a pneumotropic disease, makes such individuals more prone to pulmonary complications and mortality. This is why a higher proportion of patients requiring admission and supplemental oxygen were amongst those with cancer-related pulmonary involvement, as reflected in previous studies [13,18,24,25]. A study in China in 2020 revealed that cancer patients with pulmonary involvement had a higher mortality rate of 39% [26]. Similar outcomes were observed in a study conducted in Italy which showed a mortality rate of 20.9% in cancer patients with pulmonary involvement [27]. The observations in multiple studies were consistent with our results regarding poor pulmonary involvement and comorbidities outcomes [24-28]. However, another study conducted in China in 2022 showed no significant increase in risk in terms of worse outcomes in cancer patients with pulmonary involvement [29].

The requirement of ventilatory support did not show a significant difference between pulmonary and non-pulmonary involvement within the cancer group. This observation is attributed to the fact that many of these patients were not deemed appropriate for aggressive resuscitative measures, including ventilatory support, due to poor performance status and pulmonary reserves, a dismal prognosis, and treatment expectations compared to the general population. The cytokine storm is still the main culprit causing severe infection and mortality in COVID-19 [7]. But it can be inferred from this study that the baseline lung reserves also strongly play a crucial role in the morbidity and mortality associated with COVID-19 [9]. In all these patients, the lung reserves were not quantified using pulmonary function tests (PFTs) due to infection control restrictions on the ground of medical staff safety, however; clinical, laboratory, and imaging parameters did suggest poor pulmonary reserves in cancer patients with pulmonary involvement as compared to the general population with COVID-19 infection. 

Furthermore, three out of five cancer deaths with lung involvement had the overall disease in remission, while the rest of the two had progressive disease. Moreover, those COVID-19 cancer deaths with no lung involvement had an attributing factor of immunosuppression due to either disease or its treatment. In contrast, only two out of six cancer patients with lung involvement who survived the COVID-19 spell had progressive disease. These findings are consistent with previous studies suggesting that immunosuppression leads to poor outcomes in these patients [30]. This further strengthens our hypothesis that the cancer disease did not contribute to death. Instead, the poor lung reserves due to pulmonary involvement and immunosuppressive state led to mortality in COVID.

The findings in this study could stand true for any other viral illness which primarily affects the lungs making cancer patients with lung involvement more prone to morbidity and mortality. Therefore, this study could provide a base for future studies and research that any pneumo-trophic viral illness in patients with poor lung reserves, mainly due to cancer-related pulmonary involvement, could be associated with poor outcomes. So, prevention or timely treatment holds grounds to reduce the morbidity and mortality associated with viral infections affecting the lungs, specifically in patients with cancerous lung involvement.

One of the study's limitations was the small number of patients with metastatic pulmonary involvement, which remains the focus of the study. However, in real-world observational studies, most of the cancer survivors are those who have a non-metastatic disease. Hence, the number of cancer patients with pulmonary involvement was less than those without. This was the best that could be achieved in a real-world observational, single-center study. The other limitation of this observational study was that the data was evaluated for patients from the first wave of the COVID-19 pandemic. At that time, little was known about the appropriate management of this condition; however, it gave us an idea of how COVID-19 could potentially behave in unvaccinated cancer patients in subsequent waves. In serial COVID-19 waves, management guidelines were developed, and appropriate management guidelines and vaccination are in place today. Therefore, the results evaluated from the above study can be further evaluated in covid-vaccinated patients who can now be managed with better treatment modalities.

## Conclusions

We conclude that cancer patients with pulmonary involvement are at higher risk of complications and death from COVID-19 compared to the non-pulmonary cancer group and the general population. The unfavorable outcomes, however, are attributed to poor lung reserves in cancer patients with pulmonary involvement rather than the cancer disease itself. Early recognition and intervention are critical in cancer patients with suspected COVID-19, especially those with pulmonary involvement.
